# Mesenchymal stem cells restore the sperm motility from testicular torsion-detorsion injury by regulation of glucose metabolism in sperm

**DOI:** 10.1186/s13287-019-1351-5

**Published:** 2019-08-24

**Authors:** Chi-Hao Hsiao, Andrea Tung-Qian Ji, Chih-Cheng Chang, Ming-Hsien Chien, Liang-Ming Lee, Jennifer Hui-Chun Ho

**Affiliations:** 1 Graduate Institute of Clinical Medicine, Taipei Medical University, #250 Wu-Hsing Street, Taipei, 110 Taiwan; 2 Department of Urology, Wan Fang Hospital, Taipei Medical University, #111, Section 3, Hsing-Long Road, Taipei, 116 Taiwan; 3 Center for Stem Cell Research, Wan Fang Hospital, Taipei Medical University, #111, Section 3, Hsing-Long Road, Taipei, 116 Taiwan; 4 Institute of Clinical Medicine, National Yang-Ming University, No.201, Sec.2, Shih-Pai Rd. Peitou, Taipei, 11221 Taiwan; 5 Department of Internal Medicine, School of Medicine, College of Medicine, Taipei Medical University, Taipei, Taiwan; 6 Division of Pulmonary, Department of Internal Medicine, Shuang Ho Hospital, Taipei Medical University, #291, Zhongzheng Road, Zhonghe District, New Taipei City, 235 Taiwan; 7 TMU Research Center of Cancer Translational Medicine, Taipei Medical University, Taipei, Taiwan

**Keywords:** Glycolysis, Infertility, Mesenchymal stem cells, Sperm motility, Testicular torsion-detorsion

## Abstract

**Background:**

Testicular torsion is an urological emergency that may lead to infertility due to ischemic injury. Promptly surgical correction by orchiopexy is the only way to avoid infertility and no effective treatment for restoration of spermatogenesis. We previously reported that mesenchymal stem cells (MSCs), through local injection upon testicular torsion-detorsion, restored the spermatogenesis without differentiation into sperm. In this study, molecular mechanisms of MSCs in regulating germ cell activity induced by testicular torsion-detorsion were investigated.

**Methods:**

Sixteen male Sprague-Dawley rats 6–8 weeks old received left testis 720° torsion for 3 h followed by detorsion with or without MSCs. Right inguinal skin incision without testicular torsion served as control. MSCs with 3 × 10^4^ cells were locally injected into left testis 30 min before detorsion. Three days after the surgery, orchiectomy was executed and the testis, epididymis, and sperm were separated to each other. Functional assessments on sperm included counting sperm amount and sperm motility, staining F-actin, and quantifying adenosine triphosphate (ATP) content. The hallmarks of glycogenesis and glycolysis in each tissue segment were measured by Western blot.

**Results:**

Testicular torsion-detorsion significantly decreased the amount of sperm, inhibited the motility, declined the F-actin expression, and reduced the content of ATP in sperm. Local injection of MSCs improved sperm function, particularly in sperm motility. With MSCs, ATP content and F-actin were preserved after testicular torsion-detorsion. MSCs significantly reversed the imbalance of glycolysis in sperm and testis induced by testicular torsion-detorsion, as evidenced by increasing the expression of phosphoglycerate kinase 2 and glyceraldehyde-3-phosphate dehydrogenase-spermatogenic, activating Akt, and increasing glycogen synthase kinase 3 (GSK3), which led to the increase in glycolysis cascades and ATP production. Human stem cell factor contributed the activation of Akt/GSK3 axis when sperm suffered from testicular torsion-detorsion-induced germ cell injury.

**Conclusions:**

Local injection of MSCs into a testis damaged by testicular torsion-detorsion restores sperm function mainly through the improvement of sperm motility and energy. MSCs reversed the imbalance of glycogenesis and glycolysis in sperm by regulating Akt/GSK3 axis. Thus, MSCs may potentially rescue torsion-detorsion-induced infertility via local injection.

## Background

Testicular torsion is an urology emergency and which threats 1/4000 of male population younger than 25 years old with infertility as a sequela of germ cell ischemic/reperfusion (I/R) injury [1]. Duration of testicular torsion and the severity of cord twisting are two key prognostic factors for sperm survival and activity. However, in a cord twisting higher than 360° plus symptom duration more than 24 h, complete or severe testicular atrophy is inevitable due to cell necrosis starting from 4 h after testicular torsion. Emergency surgical detorsion by reduction and fixation within 6 h is the only way to reduce the rate of permanent dysfunction on the testis. However, surgical detorsion-induced reperfusion injury may occur, and germ cells cannot be regenerated by the surgery [1].

In our series studies, animal model with severe cell necrosis in testis was created by a 720° testicular torsion for 3 h. We previously explored that local injection of mesenchymal stem cells (MSCs) 30 min before surgical detorsion was applicable to prevent testicular torsion-induced I/R injury on germ cell and maintain the spermatogenesis at acute stage. The therapeutic effect of MSCs came from paracrine effect to inhibit testicular apoptosis, reduce intra-testicular oxidative stress, and maintain serum testosterone level. MSC-secreted stem cell factor (SCF) played a role in promoting spermatogenesis in the recipient [2].

Spermatogenesis is the process to produce the sperm termed spermatozoa from spermatogonial stem cells [3]. Human sperm cell is a flagellate cell consisting of a disc-shaped head and a long tail released from the seminiferous tubule and mature in the epididymis. An enzyme-rich cap covering the disc facilitates fertilization. There are dense fibers in the tail controlling sperm movement, and the midpiece connecting sperm tail and head is rich in mitochondria to provide energy during sperm motility [4].

In clinic, a seminogram contains sperm count, motility, morphology, volume, color, fructose level, pH value, and liquefaction [5]. Among the above parameters, total motile spermatozoa, which refers to the concentration of mature sperm with high motility, serves as the predictive factor for pregnancy [6, 7]. Using a histopathological scoring, we previously demonstrated that MSCs kept the mean value of Johnsen’s score above 8 points after testicular torsion-detorsion (8.33 ± 1.13 with MSCs vs. 6.04 ± 0.62 without MSCs) [2]. However, the impact of MSCs on the sperm quantity as well as sperm quality related to male fertility need to be further investigated.

This time, we aim to study the effect and mechanism of MSCs on sperm which suffered from testicular torsion-detorsion injury; thus, the study time point should be within 5–7 days after testicular torsion since the lifetime of mature sperm is 5–7 days in the seminiferous tubule [8]. To avoid the mature sperm storage in seminiferous tubule prior to testicular torsion masked the effect from MSCs, we performed the sperm assessments 3 days after torsion-detorsion injury. We hypothesizes that MSCs not only promotes the spermatogenesis, but also supports sperm with a better quality. The same to our previous study design, rat model of surgical unilateral 720° testicular torsion for 3 h followed by reduction was performed under general anesthesia. Local injection of MSCs was given 30 min before surgical reduction. Parameters for sperm quantity and quality including numbers, morphology, and motility were evaluated. We also explored the mechanism of MSCs on sperm against the testicular torsion-detorsion-induced germ cell injury.

## Materials and methods

### Animals

Male *Sprague-Dawley* rats at 5–7 weeks old were purchased from (BioLASCO Taiwan Co., Ltd., Taipei, R.O.C.). The rats were housed in a temperature of 24 ± 3 °C and 12-h light-dark cycle. The animals were fed with standard pellet diet and water ad libitum. Rats received surgical torsion-detorsion at the age of 6–8 weeks old after a 7-day period of acclimatization.

### Isolation and culture of MSCs

**Fig. 1:**
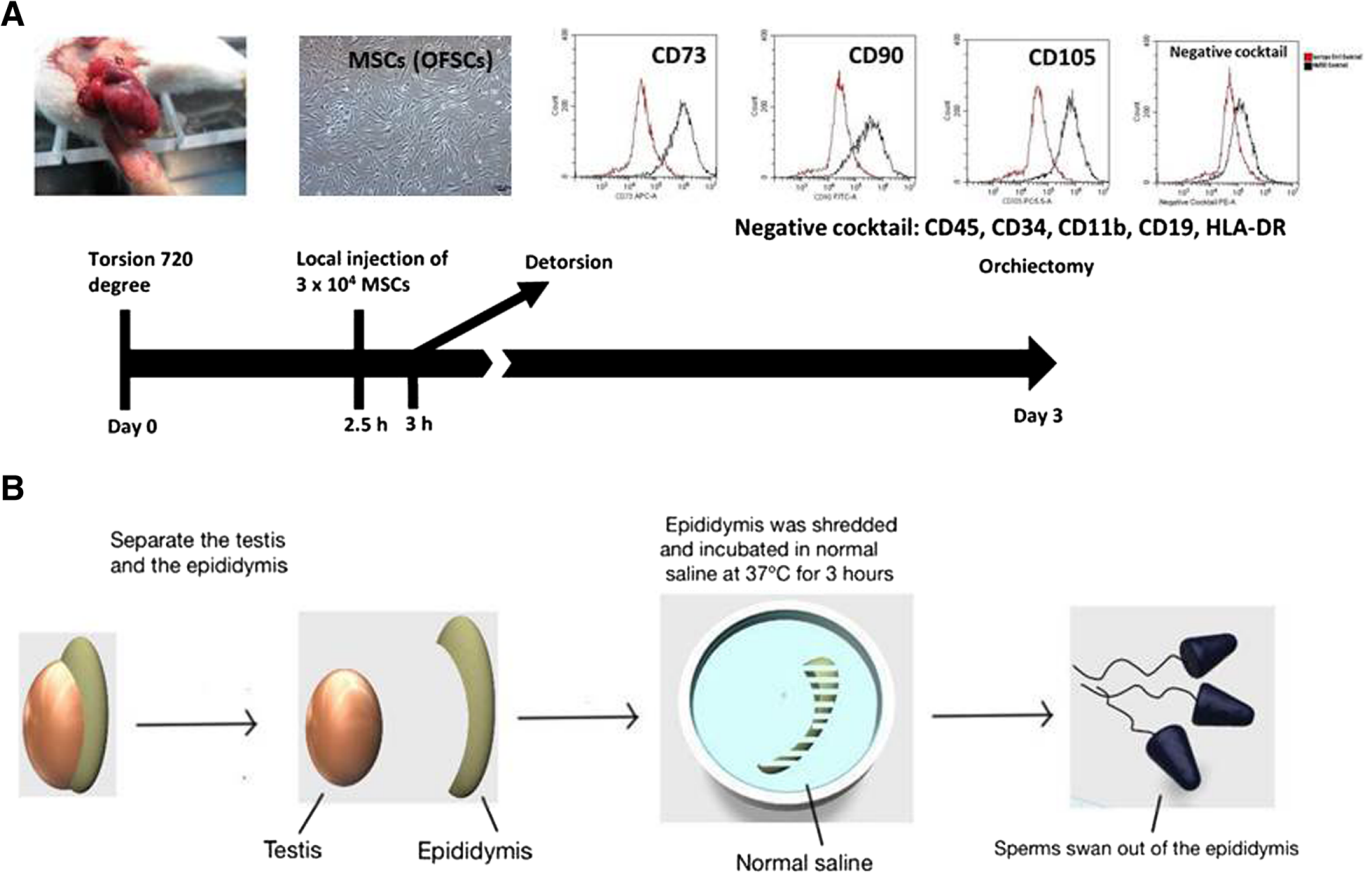
Flowchart of testicular torsion-detorsion and study design. **a** Rats received surgical 720° testicular torsion for 3 h on the left testis and sham operation of right side. Twenty microliter phosphate-buffered saline with or without 3 × 10^4^ human mesenchymal stem cells (MSC) was administrated via local injection to the left testis 30 min before detorsion. Animals were sacrificed for orchiectomy of both testis on the day 3. **b** Testis and epididymis were separated, and epididymis was shredded and incubated in normal saline at 37 °C for 3 h to collect sperm

MSCs were isolated from human orbital fat tissue (orbital fat stem cells, OFSCs) as described previously [2]. All samples were collected with the written informed consent of the subjects regulated by the Institutional Review Board of TMU-Wan Fang Hospital. Briefly, adipose tissues removed from orbital cavity were fragmented, digested, and filtered. After centrifuging the fluid, cells from the resulting pellet were plated in non-coated tissue culture flasks (BD Biosciences, Franklin Lakes, NJ, USA) and maintained in Mesen Pro Medium (Invitrogen, Carlsbad, CA, USA). MSC characteristics were demonstrated by fibroblast morphology, surface phenotyping (positive for CD29, CD90, and CD105 and negative for hematopoietic markers such as CD31, CD34, CD45, CD106), tri-lineage differentiation capacity, and immunomodulatory ability [9]. MSC phenotyping was double confirmed with human MSC analysis kits (Material No. 562245, BD Biosciences) and is illustrated in Fig. 1a.

### Experimental protocol

The experimental protocol has been approved by the Ethical Committee on Animal Research at Wan Fang Hospital. SD rats were randomized into 2 groups, as (1) torsion-detorsion group (T): animals received surgery of left testicular torsion and detorsion (*N* = 7); (2) torsion-detorsion with MSCs treatment (T + MSC): animals received operation of left testicular torsion and local injection of MSCs into left testis before detorsion (*N* = 9). Each animal received sham operation (surgical inguinal skin incision on the right side without testicular torsion) as control (Ctrl, *N* = 16).

All surgical procedures were performed under anesthesia as previous description [2]. Briefly, unilateral testicular torsion was performed by a 720°, clockwise rotation on the left testis followed by hemiscrotum fixation with 4–0 atraumatic silk suture. Three hours later, the spermatic cord was detorsed and MSCs was injected locally into left testis 30 min before detorsion. After 3 days, orchiectomy was performed and tissue samples were divided into testis, epididymis, and sperm, respectively.

### MSC transplantation

In our previous study, local injection of MSCs (OFSCs) with 3 × 10^4^ cells in 20 μl phosphate-buffered saline (PBS) demonstrated a therapeutic effect against testicular torsion-detorsion injury [2]. This time, the same concentration of MSCs was injected into testis in T+MSC group. Local injection of 20 μl PBS into testis served as placebo control to T group. According to experiments from previous studies, the potential application of OFSCs in acute lung injury [10], type 2 diabetes [11], corneal injury [12], and testicular torsion-detorsion injury [2] have been demonstrated. The optimal therapeutic dosage of MSCs (OFSCs) for transplantation was around 3 × 10^7^ cells/kg bodyweight [10, 11]. In the previous study, local injection of 3 × 10^4^ MSCs (OFSCs), based on the weight of one testis, in 20 μl PBS, has been demonstrated to be able to protect testis from testicular torsion-detorsion induce injury [2]. This time, the same dose was chosen to investigate effect on sperm protection.

### Sperm amount and mobility

After sacrifice, rat’s epididymis was removed, minced, and incubated in normal saline at 37 °C for 2 h to extricate the sperm. According to the report from the World Health Organization, among the assessments on sperm quality, the ability of male fertility is determined by sperm morphology, sperm count (number), and sperm motility [6, 7]. The ability of sperm motility can be quantified by the number of sperm with vitality (movable sperm) and progressive movement (sperm with the ability to move forward). Morphology of sperm, number of sperm, and the percentage of movable sperm as well as sperm moving forward were counted under microscopy.

### Western blot analysis

After sacrifice, central testis and epididymis were cut off and sliced into small pieces, and then a piece about 0.5 mg was minced and lysed in tissue lysis buffer immediately for protein purification. Sperm extracts were lysed, and the protein sample purified from the cell lysates was prepared. Protein samples were prepared according to a previously described protocol [10–12]. Thirty micrograms of protein from each sample was separated on 10% SDS-PAGE and blotted onto PVDF membrane (Amersham Biosciences, Uppsala, Sweden), followed by blocking with 5% skim milk in TBST buffer (50 mM Tris-HCl, 150 mM NaCl, 0.1% Tween 20, pH 7.4). The membrane was then blotted with indicated primary antibody against phosphoglycerate kinase 2 (PGK2) (1:1000, Cat. No.sc-48342, Santa Cruz, Dallas, TX, USA), glyceraldehyde-3-phosphate dehydrogenase-spermatogenic (GAPDHS) (1:1000, Cat. No.sc-25778, Santa Cruz), phosphorylated Akt (1:500, Cat. No. ab38449, Abcam, Cambridge, MA, USA), AKT (1:500, Cat. No. ab106693, Abcam), phosphorylated GSK3 (1:500, Cat. No. ab68476, Abcam), GSK3 (1:1,000, Cat. No. ab62368, Abcam), human SCF (1:10,000, Cat. No.ab52603, Abcam), or human insulin-like growth factor 1 (IGF-1, 1:1,000; Abcam, Cat. No.ab106836) followed by secondary antibodies against the fragment crystalizable region of primary antibodies. Cyclophilin (1:2000, Cat. No. ab16045, Abcam) or β-actin (1:10,000, Cat. No. A5441, Sigma, St. Louis, MO, USA) was used as internal control. The density of protein bands were assessed using a computing densitometer with Image-Pro plus software (Media Cybernetics, Inc., Rockville, MD, USA).

### Detection of F-actin expression in sperm

Sperm cells were smeared on slides and fixed with 4% paraformaldehyde. The F-actin in sperm was stained with Phalloidin–Tetramethylrhodamine B isothiocyanate (Sigma,) and counterstained with DAPI.

### Adenosine triphosphate (ATP) content

ATP content was measured with ATP Colorimetric/Fluorometric Assay Kit (BioVision, Milpitas, CA, USA). A hundred thousand of sperm cells were lysed in 100 μl ATP assay buffer. The lysed sample was mixed with kit reagent in a 96-well plate, then incubated at room temperature for 30 min away from light. The intensity of absorbance at 570 nm was proportioned to the ATP level.

### Statistical methods

Each study method was performed at least three independent experiments in each group. Values are expressed as the mean ± standard deviation. Statistically significant differences between treatment group and control group were assessed by one-way analysis of variance followed by a Tukey–Kramer *t* test using Prism 6.1d (GraphPad Software, Inc., La Jolla, CA, USA), and differences were considered statistically significant with probability (*p*) < 0.05 for quantifying Western blot analysis and *p* < 0.001 for sperm quality analysis and ATP content measurement.

## Results

### Experimental study design on sperm analysis

Animal study design is illustrated in Fig. 1a. Animal model of torsion-detorsion-induced testicular ischemic reperfusion injury was the same to our previous study published in 2015 [2]. In the previous study, paracrine effect of MSCs by testicular injection was able to rescue spermatogenesis. This time, to study the paracrine effect of MSCs on sperm quality upon testicular torsion-detorsion injury, rats were divided into three groups, i.e., Ctrl, T, and T+MSCs groups. Prior to MSC transplantation, MSC phenotype was double confirmed. As illustrated in Fig. 1a, cells expressed MSC markers including CD73, CD90, and CD105 and were negative for CD45, CD34, CD11b, CD19, and HLA-DR. Following, we performed sham operation without torsion to the right testis of rats as control (Ctrl). The rats in torsion-detorsion group (T) received surgical testicular torsion of left testis for 3 h and then detorsion. Rats in torsion-detorsion with MSCs treatment group (T+MSC) received testicular torsion of left testis for 3 h and local injection of MSCs 30 min before detorsion. On the 3rd day after surgery, orchiectomy was performed to isolate both testis and epididymis. Testis and epididymis were separated to different discs and with warm PBS. Epididymis was further sliced to facilitate sperm extraction (Fig. 1b).

### Improvement of testicular torsion-detorsion-induced sperm defect by MSCs

**Fig. 2:**
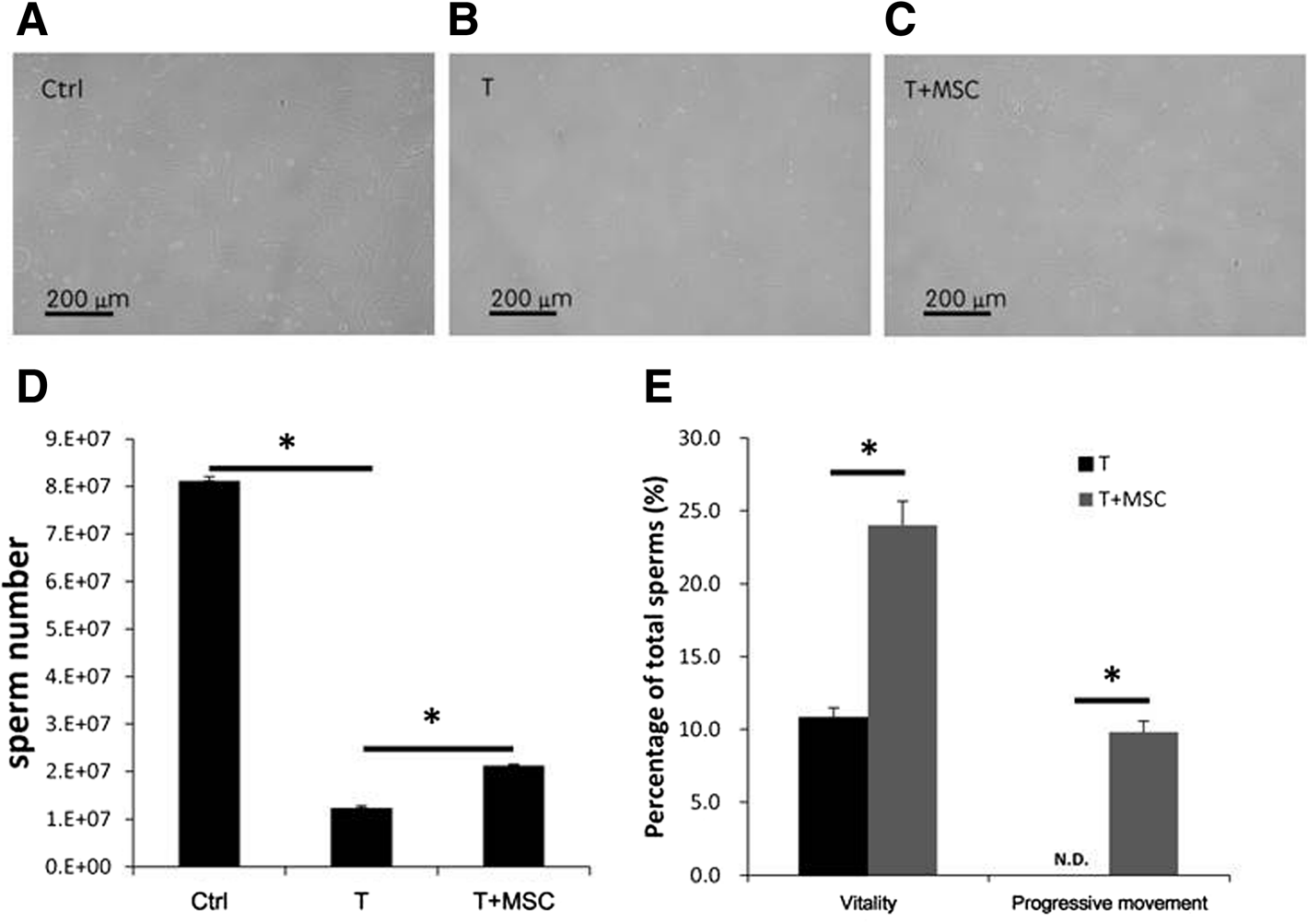
Improvement of sperm function by MSCs. The morphology of sperm in **a** sham operation (Ctrl), **b** torsion-detorsion (T), and **c** torsion-detorsion with MSC (T+MSC) groups were similar. MSCs increased sperm number (**d**) and mobility (**e**) affected by testicular torsion-detorsion. Analysis of variance with the Tukey *t* test. *p < 0.001, *n* > 6 in each group

We first evaluated the effect of MSCs on sperm quality against testicular torsion-detorsion injury. Figure 2 shows the morphology, number, and mobility of sperm from rats with different groups. Testicular torsion-detorsion did not obviously affect the morphology of sperm (Fig. 2a–c); however, the sperm number was significant (*p* < 0.001) decreased in the testicular torsion-detorsion group (T) (Fig. 2d). Although MSC treatment (T+MSC) did not fully rescue testicular torsion-induced sperm cell loss, the treatment of MSCs significantly (*p* < 0.001) improved the sperm number compared to that in the torsion-detorsion group (T) (Fig. 2d). Clinically, the number of sperm with vitality (movable sperm) and number of sperm with progressive movement (sperm with the ability to move forward) represent the ability of sperm motility that is critical for male fertility [6, 7] . It is observed that only few of movable sperm and no sperm with progressive movement in torsion-detorsion group (Fig. 2e, black bar). MSC treatment not only significantly increased the vitality, but also markedly improved the progressive motility of sperm of rats with testicular torsion-detorsion (Fig. 2e, gray bar). These data suggested that MSC administration significantly (*p* < 0.001) recovered sperm production and sperm vitality after testicular torsion-detorsion.

### MSC restoration of energy to support sperm motility

**Fig. 3:**
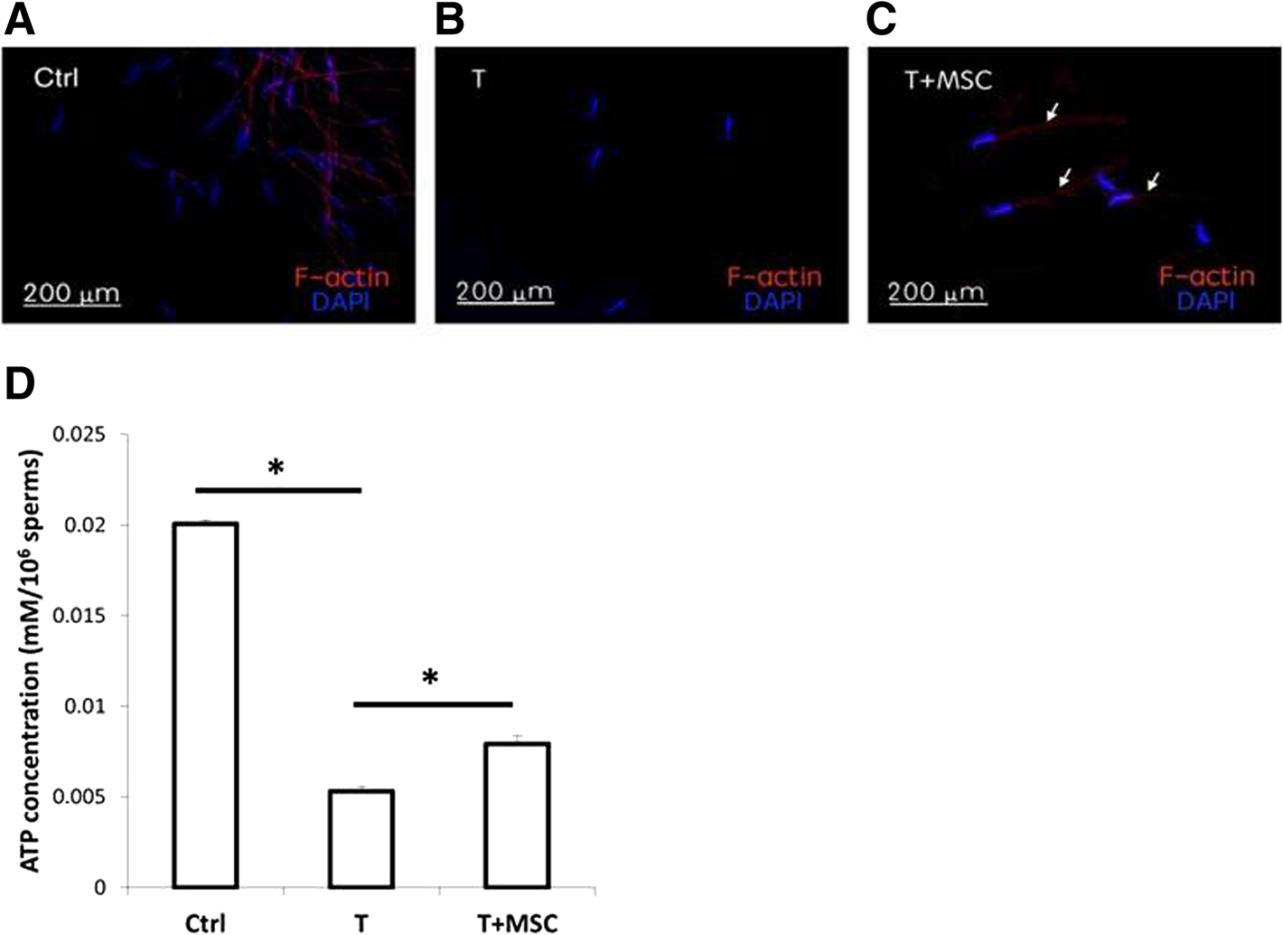
Restoration of sperm energy by MSCs. Complete loss of F-actin at sperm tail by testicular torsion-detorsion and some of F-actin preserved by MSCs were noted compared with the fluorescent stained for F-actin in sperm from **a** control, **b** torsion-detorsion (T), and **c** torsion-detorsion with MSC (T+MSC) groups. **d** With MSC treatment, amount of ATP in sperm was increased in a sperm suffering from torsion-detorsion injury. Analysis of variance with the Tukey *t* test. *p < 0.001, *n* = 3 in each group

F-actin is essential for mammalian sperm flagellar motility [13]. With sham operation (Ctrl), F-actin was strongly detectable in the sperm midpiece and tail (Fig. 3a). As shown in Fig. 3b, F-actin signal was absent in the sperm after testicular torsion-detorsion (T). With MSC treatment (T+MSC), F-actin was partially restored in the sperm midpiece and tail after testicular torsion-detorsion (Fig. 3c). In addition to the amount of F-actin, energy support is critical for sperm movement. We measured the ATP content of sperm in Ctrl, T, and T+MSCs groups, respectively. Data in Fig. 3d demonstrate that testicular torsion-detorsion blocked ATP production in sperm while MSC treatment significantly increased the amount of ATP in sperm (*p* < 0.001) compared to that in the torsion-detorsion group (Fig. 3d).

### MSC restoration of energy by promoting glycolysis in sperm

**Fig. 4:**
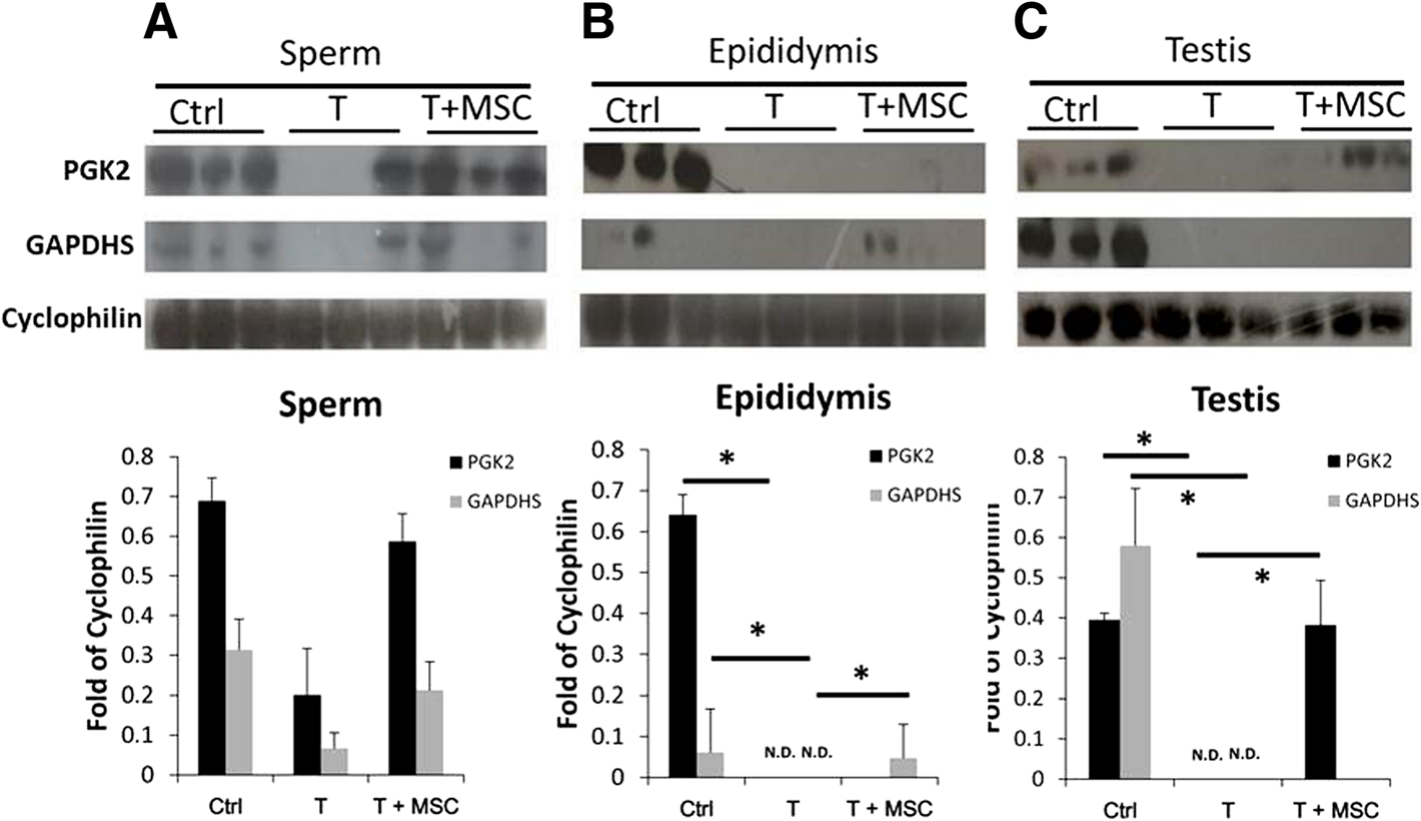
Improvement of glucose metabolism by MSCs. Measurement of PGK2 and GAPDHS in **a** sperm, **b** epididymis, and **c** testis, respectively, demonstrated that MSCs improved the dysfunction of glycolysis in sperm and in testis caused by testicular torsion-detorsion. The intensity of target protein was normalized to housekeeping protein cyclophilin. Analysis of variance with the Tukey *t* test. *p < 0.05, *n* = 3 in each group

In a sperm, the amount of ATP for sperm flagellation is regulated by dynamic balance on glycogenesis and glycolysis [14]. The more active on glycolysis or the less active on glycogenesis results in increased ATP and enhancement of sperm motility. In the present study, the expression of PGK2 and GAPDHS that serve as two marker indicators for glycolysis [15] and the effect of MSCs on the glucose metabolism in sperm (Fig. 4a), epididymis (Fig. 4b) and testis (Fig. 4c) from testicular torsion-detorsion rats were evaluated, respectively. Compared to the sham operation (Ctrl), the protein level of PGK2 in sperm was occasionally detectable (Fig. 4a) and in epididymis and testis were undetectable (Fig. 4b, c) after testicular torsion-detorsion (T). PGK2 was restored in sperm and testis by MSC administration (T + MSCs) (Fig. 4a, c). Similar to PGK2, the protein level of GAPDHS in sperm was occasionally detectable (Fig. 4a) and in epididymis and testis was undetectable (Fig. 4b, c) in the torsion-detorsion group (T), which was partially recovered by MSCs treatment (T+MSCs) in sperm and epididymis (Fig. 4a, b).

### Modulation of Akt/GSK3 axis by MSCs

**Fig. 5:**
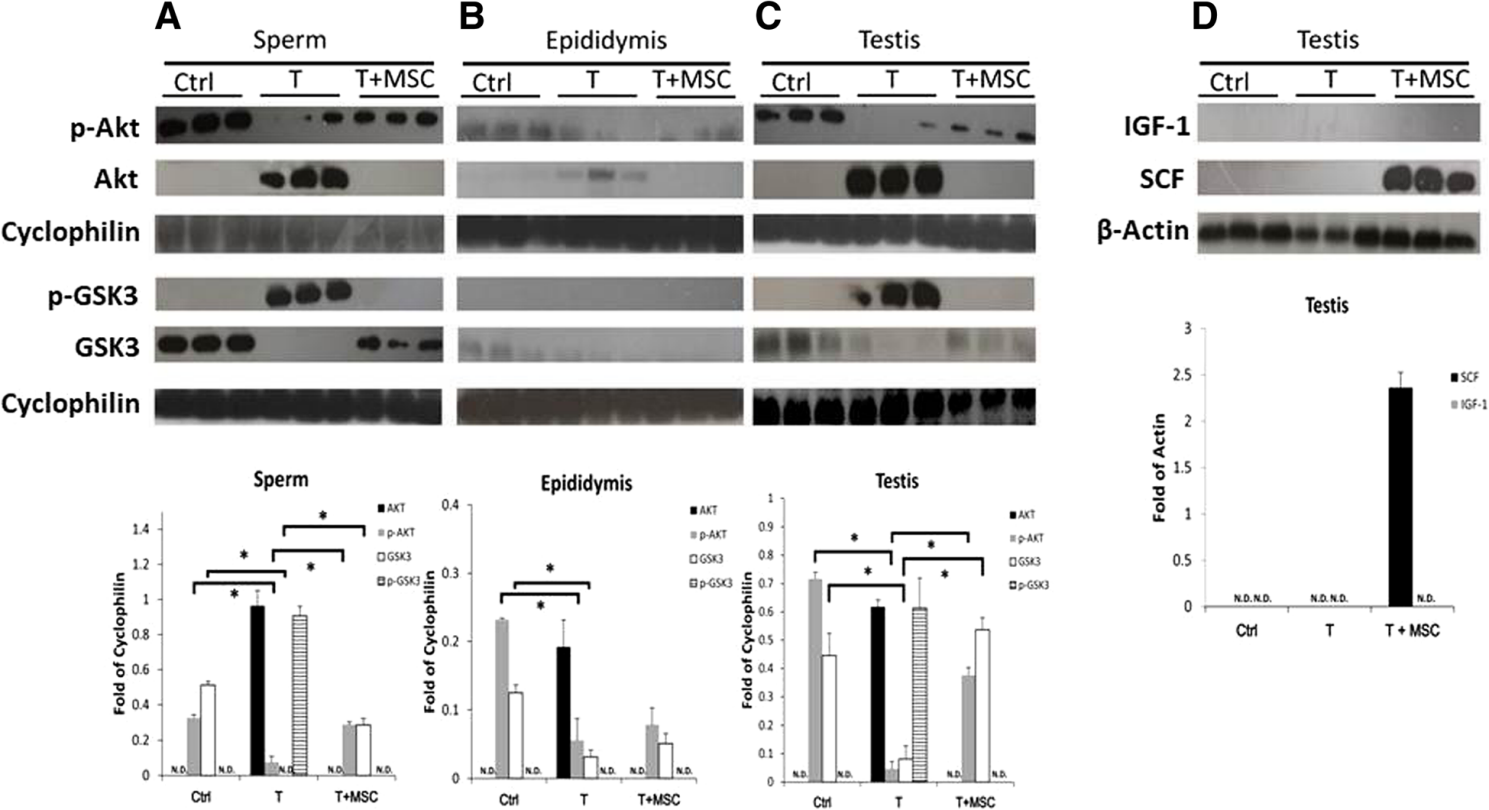
Activation of Akt/GSK3 axis by MSCs modulates the expression of Akt and GSK3 expression of testicular torsion-detorsion-treated rats by secretion of stem cell factor. Amount of Akt, GSK3, and their phosphorylated forms in **a** sperm, **b** epididymis, and **c** testis revealed that MSCs reversed the inactivation of Akt/GSK3 caused by testicular torsion-detorsion, particularly in sperm and in testis. **d** Human stem cell factor (SCF) but not human insulin growth factor-1 (IGF-1) was detectable in testis of testicular torsion-detorsion (T)-treated rats treated with MSCs. Analysis of variance with the Tukey *t* test. *p < 0.05, *n* = 3 in each group

Akt/GSK3 axis controls the balance of glycogenesis/glycolysis in sperm [16]. GSK3 is a multi-tasking kinase related to many cellular processes such as glycolysis, metabolism, signal transduction, apoptosis, and cell cycle regulation [17]. The function of GSK3 is to inhibit glycogen synthesis resulting in promotion of glycolysis. GSK phosphorylation is unfavorable to glycolysis. Akt activation (phosphorylated Akt) enhances the effect of GSK3 by inhibiting GSK3 phosphorylation. In the present study, the regulatory effect of MSCs on Akt/GSK3 axis in sperm (Fig. 5a), epididymis (Fig. 5b) and testis (Fig. 5c) in rats with the treatment of testicular torsion-detorsion was assessed, respectively. Compare to sham operation control (Ctrl), testicular torsion-detorsion resulted in loss of phosphorylated Akt (p-Akt) in sperm, epididymis, and testis, which was accompanied by accumulation of Akt in sperm, epididymis, and testis (Fig. 5a–c). With MSC treatment (T+MSC), similar to the results in control group, Akt was no longer accumulated in sperm, epididymis, and testis, and p-Akt were significantly increased in the sperm and testis compared to p-Akt in the torsion-detorsion group (T) (Fig. 5a, c). On the other hand, protein level of GSK3 can be strongly detected in sperm and testis (Fig. 5a, c) but less protein signals in epididymis (Fig. 5b). GSK expression was significantly affected by testicular torsion-detorsion (Ctrl vs. T) in all tissue fractions (Fig. 5a–c), which associated with phosphorylated GSK3 accumulation in sperm and in testis (Fig. 5a, c). MSCs significantly increased GSK3 expression in sperm and testis under torsion-detorsion injury, similar to the results in the control group; p-GSK3 was no longer accumulated in the sperm and testis with MSC treatment (Fig. 5a, c).

### Paracrine SCF from MSCs attributing the sperm protection

Akt/GSK3 axis is the downstream signal of ligand/receptor interaction of SCF/c-Kit. SCF conjoined with c-Kit receptor phosphorylates AKT resulting in decreased GSK activity [18]. As shown in Fig. 5d, the human SCF was only detectable in the testis with MSC treatment (T+MSC) and was undetectable in epididymis and sperm. Same to our previous finding, human IGF-1 cannot be found in testis, epididymis, and sperm tissue after MSC treatment. These findings implied that human SCF from MSCs may trigger the signals via c-Kit activation in the testis, including sperm in the testis.

## Discussion

**Fig. 6:**
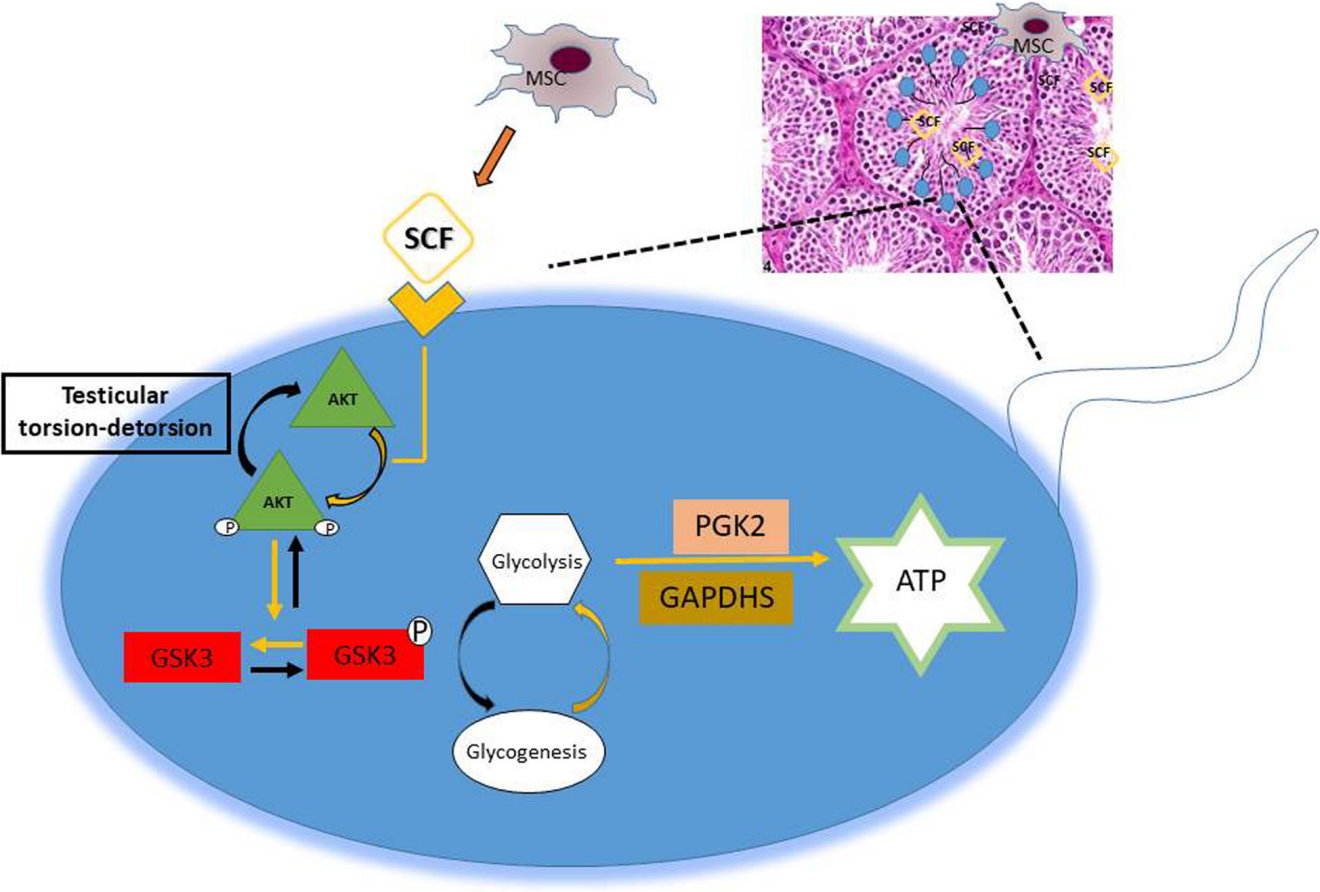
Schematic illustration mechanisms of sperm motility restoration by MSCs upon testicular torsion-detorsion. Testicular torsion-detorsion severely affected sperm motility resulted from loss of ATP, while testicular injection of MSCs increased ATP content in sperm and enhanced sperm mobility by promoting glycolysis process and regulating Akt/GSK axis

As illustrated in Fig. 6, data from this study demonstrated that sperm number and sperm motility were significantly affected by testicular torsion-detorsion. MSC injection before surgical detorsion helped with restoration of sperm quality, particularly in sperm motility (Fig. 2) evidenced by positive F-actin signals in sperm tail and increased ATP content in sperm (Fig. 3). Testicular torsion-detorsion abrogated glycolysis in sperm, epididymis, and testis by markedly reduction of PGK2 and GAPDHS, and MSCs significantly improved the glycolysis in sperm and testis (Fig. 4). After testicular torsion-detorsion, levels of Akt and p-GSK3 were dramatically increased while levels of pAkt and GSK3 were difficult to be detectable in sperm and in testis. However, the imbalance of Akt/GSK3 axis induced by testicular torsion-detorsion was reversed by MSCs and that was associated with paracrine effect of human SCF from MSCs in the testis (Fig. 5).

The incidence of testicular torsion is relatively higher in newborns, children, and adolescent boys than in adults. The sequelae of testicular torsion and surgical detorsion, i.e., infertility, is majority due to I/R injury from the increased reactive oxygen species (ROS) [19]. Although supplementation of antioxidants such as *N*-acetylcysteine and ethyl pyruvate significantly reduces ROS level [20], deficient spermatogenesis is not able to be reconstructed by antioxidants. It was reported that uncultured adipose-derived stromal vascular fraction showed protective effects on testicular torsion-detorsion-induced oxidative damage in Leydig and germ cells and promoted spermatogenesis [21]. In the present study, we demonstrated that adipose-derived MSCs restored sperm function upon testicular torsion-detorsion-induced germ cell injury.

Clinically, infertility is determined by the measurements of sperm morphology, concentration, and motility. In most male infertility studies, chemical-induced depletion of sperm was a common animal model and bone marrow-derived MSCs (BM-MSCs) were commonly used for the assessment of the role of these cells in infertility therapy. BM-MSC paracrine supported the cell proliferation, prevented apoptosis, and secreted growth factors such as retinoic acid, bone morphogenetic protein (BMP) 4, BMP8b, and transforming growth factor beta1 which could support the differentiation ability of BM-MSCs into germ-like cells [22, 23]. In our series study, we established male infertility animal model via surgical torsion-detorsion. Orbital fat-derived MSCs (OFSCs), through diminishing apoptosis and oxidative stress as well as increasing testosterone secretion, protect testis from torsion-detorsion injury. However, MSCs did not possess the ability of germ cell differentiation in the acute stage after testicular torsion-detorsion [2]. In the acute stage, paracrine support from MSC-secreted SCF not only restored the spermatogenesis [2] but also preserved sperm function mainly through improvement of sperm motility by regulation of glucose metabolism (Figs. 2, 3, 4, and 5).

Conditioned medium from human dental pulp stem cells or human bone marrow-derived MSCs showed protective effects against ischemic-induced damage by decreasing the ROS production and inflammatory reaction and further recovered the cellular ATP content in human astrocytes [24]. When MSCs co-culture with islet cells, MSCs were able to enhance the mRNA level of glucose transporter 2 and to restore energy in islet cells [25]. In germ cells, with MSCs, the integrity and the vitality of sperm structure was recovered as evidenced by the increased level of F-actin and ATP content when suffering from testicular torsion-detorsion-induced germ cell injury (Fig. 3).

Effect of I/R damage on glycolysis depends on the cell/tissue types. In cardiomyocytes, short-time hypoxia induces glycolysis but long-time hypoxia suppresses glycolysis [26]. In renal tissue, bilateral renal ischemia-induced metabolic modulation occurs as early as 6 h post reperfusion with significant induction of gluconeogenesis. Glycolysis pathway is dominant at 48 h after reperfusion [27]. In testis tissue, we first found that testicular torsion-detorsion induced glycogenesis and reduced glycolysis in sperm, epididymis, and testis, while MSC treatment significantly reversed the imbalance of glycogenesis/glycolysis in sperm and testis (Figs. 4 and 5).

It is well known that glycolysis is the way to produce ATP in sperm and crucial for sperm motility [14]. PGK2 and GAPDHS are common enzyme markers to determine the activity of glycolysis [15]. Glycolysis in sperm is regulated by phosphoinositide 3-kinase (PI3K)/Akt/GSK3 axis [16], and exosomes derived from MSC have been reported to activate PI3K/Akt signaling in human dermal fibroblast [28]. It was observed that testicular torsion-detorsion shot down the glycolysis in sperm, epididymis, and testis. Glycolysis activities were significantly affected by testicular torsion-detorsion in sperm, epididymis, and testis (Fig. 4a–c), and MSCs stored the glycolysis, after suffering from torsion-detorsion injury, of sperm (Fig. 4a) and testis (Fig. 4c).

It has been reported that inhibition of GSK3 stimulates glycogen synthase and glucose transport in adipocytes [29]. Recently, MSC secretome components were found to restore spermatogenesis in a rat model of bilateral abdominal cryptorchidism [30]. In our study, testicular torsion-detorsion injury significantly inhibited the activity of Akt/GSK3 axis in sperm, epididymis, and testis by accumulation of Akt and p-GSK3 (Fig. 5a–c). We showed that MSC treatment reactivated Akt/GSK3 axis, particularly in sperm and testis after testicular torsion-detorsion by shifting Akt and p-GSK3 to be p-Akt and GSK3 (Fig. 5a, c).

SCF is a ligand of c-Kit to control the axis of PI3K/Akt/GSK3 axis, and SCF upregulates cyclin D3 via the PI3K/p70 s6 kinase pathway in spermatogonia [31]. In the testis, SCF is produced by Sertoli cells and is an activator of spermatogonial proliferation. Furthermore, c-Kit (or truncated tyrosine kinase receptor) and SCF are suggested as biomarkers for the evaluation of human sperm quality [32]. It has been reported that SCF/c-Kit pathway plays an anti-apoptotic role in modulating inflammation and ROS regulation [33]. In our previous study, SCF from MSCs was highly expressed in a testis with torsion-detorsion injury, indicating that spermatogenesis is contributed by MSC-secreted SCF supporting germ cell proliferation and against apoptosis [2]. Moreover, the sperm maturity and vitality may also be improved by SCF/c-Kit interaction resulting in the activation of Akt and decrease in GSK activity [18]. Via testicular injection of MSCs, human SCF was only found in the testis, but not in epididymis and sperm (Fig. 5d), which implied the SCF in testis may not only trigger the spermatogenesis and sperm maturation, but also preserve sperm quality from testicular torsion-detorsion-induced germ cell injury.

Data from this study supported that MSCs improved the number and motility of sperm with testicular torsion/detorsion injury within 3 days, which particularly helped with sperm motility. However, optimal dose of MSCs for recovery of sperm motility and sperm number as well as therapeutic effect of MSCs on long-term spermatogenesis and potential sequela affected on contralateral testis by antisperm antibody after testicular torsion need to be further investigated.

## Conclusion

In summary, MSC treatment preserves sperm quality and recovers the imbalance of glycogenesis/glycolysis induced by testicular torsion-detorsion. The therapeutic benefit of MSCs on sperm motility is contributed by activation of Akt/GSK3 axis and promotion of glycolysis that results in increasing content of ATP in sperm. Data from this study suggested that paracrine SCF from MSCs is associated with regulation of sperm motility.

## Data Availability

All data generated and/or analyzed during this study are available from the corresponding author upon reasonable request.

## Abbreviations

ATP: Adenosine triphosphate
GAPDHS: Glyceraldehyde-3-phosphate dehydrogenase-spermatogenic
GSK3: Glycogen synthase kinase 3
I/R: Ischemic/reperfusion
IGF-1: Insulin-like growth factor 1
MSC: Mesenchymal stem cell
OFSC: Orbital fat stem cell
PBS: Phosphate-buffered saline
PGK2: Phosphoglycerate kinase 2
PI3K: Phosphoinositide 3-kinase
ROS: Reactive oxygen species
SCF: Stem cell factor
